# Imaging of pancreatic metastases from renal cell carcinoma

**DOI:** 10.1186/1470-7330-14-5

**Published:** 2014-04-22

**Authors:** Matteo Vincenzi, Giulio Pasquotti, Roberta Polverosi, Claudio Pasquali, Fabio Pomerri

**Affiliations:** 1 Dipartimento di Medicina, Istituto di Radiologia, Università degli Studi di Padova, via Giustiniani 2, 35128 Padova, Italy; 2 UOC Radiologia, Ospedale di S. Donà di Piave (Ve), ULSS10 Regione Veneto, Italy; 3 Azienda Ospedaliera di Padova, Dipartimento di Scienze Mediche e Chirurgiche, Clinica Chirurgica IV, via Giustiniani 2, 35128 Padova, Italy; 4 Oncological Radiology Unit, Veneto Istitute of Oncology IOV-IRCCS, via Gattamelata 64, 35128 Padova, Italy

**Keywords:** Metastases, Pancreas, Renal cell carcinoma, Computed tomography, Magnetic resonance imaging

## Abstract

**Background:**

To describe the main imaging characteristics of pancreatic metastases from renal cell carcinoma (RCC) with particular attention to CT features, underlining possible criteria for a differential diagnosis.

**Methods:**

15 patients have been included in this study. 14 patients underwent multislice CT with triphasic acquisition (unenhanced, pancreatic parenchymal and portal venous phases). In 9 cases a delayed phase (120 sec) was also acquired. 5 patients underwent MRI, before and after administration of gadolinium.

**Results:**

The mean time interval between nephrectomy and recurrence was 7.5 years (range 1-17 years). On CT metastases avidly enhanced in the parenchymal phase and then demonstrated a significant wash-out, approaching isodensity to the normal pancreatic parenchyma in the portal phase. In the portal phase 20 of the 25 lesions found in the arterial phase were recognizable. On non-enhanced scans, only 13 of the 25 lesions were detected.

On MRI, with the limitations due to the paucity of cases, the metastatic foci appeared hypointense to normal pancreatic tissue on T1-weighted images, and hyperintense on T2- and diffusion-weighted images. After gadolinium, the behaviour was similar to that reported for CT, except for one patient in whom two metastatic foci presented a signal intensity almost isointense to the surrounding parenchyma, accompanied also by an unusual lowering of the signal on DWI (diffusion-weighted imaging) with high b-values. Compared to CT, with MRI the lesions appeared all detectable even on non-enhanced acquisitions.

**Conclusion:**

Renal Cell Carcinomas require a prolonged CT or MRI follow-up.

In patients with RCC history, an early arterial or a pancreatic parenchymal phase is always mandatory, as pancreatic metastases typically present themselves as hypervascular lesions. This behavior is similar to that of neuroendocrine tumors, while the other primary pancreatic tumors tend to be hypovascular.

## Background

Metastatic lesions in the pancreas are uncommon and account for 2% to 5% of all pancreatic malignancies [1-3]. However, pancreas is a possible site for metastases from renal cell carcinoma (RCC), not unfrequently being the only metastatic site [4].

Very delayed metastases, i.e. later than ten years after tumor nephrectomy, occur in more than 10% of patients [5].

Metastases are only rarely symptomatic in the early phases, with symptoms including abdominal pain, back pain, gastrointestinal bleeding due to duodenal infiltration, obstructive jaundice, weight loss, pancreatitis, and diabetes.

Retrospective series showed that when there is no evidence of metastatic spread in other organs, pancreatic metastatic lesions from RCC are a favorable indication for a radical surgery, offering good chances of long-term survival [6-8]. In a recent study Karam JA et al. reported that metastasectomy is feasible with acceptable morbidity in a cohort of select patients with a limited tumor burden after targeted therapy [9]. Moreover, it has been demonstrated that tumor burden characteristics are associated with clinical outcome in patients with metastasis from RCC treated with vascular endothelial growth factor-targeted therapy such as sunitinib [10].

For these reasons, it has been suggested that patients with a history of RCC should be monitored in order to detect early recurrences. Efficacy and modalities of post-operative radiological follow-up are still debated.

In this article, we describe the main imaging features of pancreatic metastases from RCC on the basis of a retrospective review of fifteen cases from our archives and a review of the literature.

## Methods

A retrospective search for patients with a diagnosis of pancreatic metastases from RCC obtained between February 2004 and June 2010 at the University Hospital of Padova was performed.

Fifteen cases were identified and their clinical records reviewed. Diagnosis was based on medical history, imaging and histological examination of surgical specimens.

The group of patients comprised six women and nine men, aged between 39 and 82, with a mean age of 63.5 years.

Fourteen out of fifteen patients underwent multislice CT, using 16- and 64-slice scanners (Emotion 16 and Somatom Sensation 64; Siemens, Erlangen, Germany). An initial non-enhanced acquisition was performed, followed by a breath-hold pancreatic parenchymal phase acquisition at 30-40 sec after injection and a portal venous phase scan, obtained 60–70 seconds after injection. In 9 cases a delayed phase of 120 sec was also acquired, even if for purposes different from the characterization of the pancreatic tumor. The intravenous administration of 150-200 mL of non-ionic contrast material (Omnipaque 350 – GE Healthcare – USA) was given using an automatic injector at a rate of 3.5-5 mL/sec.

Four-millimeter axial images, as well as coronal and sagittal reformatted images, were sent to the picture archiving and communication system.

Five patients underwent an abdominal 1.5 Tesla MRI scan (Magnetom Espree, Siemens, Erlangen, Germany) (one only MRI, four CT plus MRI) before and after i.v administration of gadolinium. Our protocol included axial GRE T1 in-phase/out-of-phase images (TR of 129 ms, TE respectively of 4.87 and 2.38 ms; field of view 380 mm; slice thick-ness, 4 mm), TSE T2 weighted images (TR, 3700 ms; TE, 92 ms; field of view 380 mm; slice thick-ness, 4 mm), axial and coronal HASTE T2-weighed images with and without fat saturation (TR, 1500 ms; TE, 90 ms; field of view, 380 mm; slice thick-ness, 4-6 mm), and diffusion-weighted (b 50-400-800 s/mm2) images.

After administration of contrast agent axial GRE T1-weighted images (TR, 5.07 ms; TE, 2.38 ms, field of view 400 mm; slice thick-ness, 4 mm) were obtained at 30, 70 and 120 seconds.

Images were evaluated by three radiologists.

Fourteen patients underwent surgery, while in one patient the diagnosis was autoptical. Both typical resections (pancreatoduodenectomy, distal splenopancreatectomy, total pancreatectomy) and atypical resections (enucleation, middle pancreatectomy, spleen-preserving distal pancreatectomy) were performed. Histological examination of the resected specimens showed metastases from renal cell carcinoma in all of these cases. All patients were treated according to the Helsinki declaration ethical principles. We obtained our hospital’s institutional review board approval to conduct a retrospective review of the patients’ medical and imaging records.

## Results

A total of 26 metastatic lesions were identified in 15 patients: 19 lesions were detected with CT, 1 with MRI and 6 with both CT and MRI (Tables 1 and 2).

**Table 1:** CT characteristics and distribution of pancreatic metastases

**Patient**	**N°**	**Diameter (mm)**	**Location**	**Unenhanced**	**N° unenh**	**Arterial**	**N° arterial**	**Venous**	**N° venous**	**Delayed**	**N° delayed**
1	2	10/18	Head/Body	Isodense	1	Hyperdense	2	Hyper/Isodense	2		
2	2	16/22	Head/Body	Hypodense	1	Hyperdense	2	Hyperdense	2	Isodense	1
3	1	40	Head	Hypodense	1	Hyperdense	1	Hyperdense	1	Hyper/Isodense	1
4	2	17/11	Head/Body	Isodense	2	Hyperdense	2	Hyper/Isodense	1	Isodense	2
5	1	10	Body	Hypodense	1	Hyperdense	1	Isodense	0		
6	3	10/5/5	Head/Body/Tail	Hypodense	1	Hyperdense	3	Isodense	2	Hypodense	1
7	2	16/8	Body/Tail	Isodense	1	Hyper/Isodense	2	Hyper/Isodense	2	Isodense	1
8	1	10	Tail	Isodense	0	Hyperdense	1	Hyperdense	1	Isodense	0
9	3	12/8/5	Head/Body/Tail	Hypodense	1	Hyper/Isodense	3	Hyper/Isodense	2		
11	1	7	Body	Hypodense	0	Hyperdense	1	Hyper/Isodense	1	Hypodense	1
12	1	39	Tail	Hypodense	1	Hyperdense	1	Hyperdense	1	Hyper/Isodense	1
13	2	16/10	Body/Tail	Hypodense	1	Hyperdense	2	Hyper/Isodense	2	Hypodense	2
14	1	20	Body	Isodense	1	Hyperdense	1	Hyperdense	1		
15	3	10/45/6	Head/Body/Tail	Hypodense	1	Hyper/Isodense	3	Hyper/Isodense	2		
Sum	25				13		25		20		10

**Table 2:** MRI characteristics and distribution of pancreatic metastases

**Patient**	**N°**	**Diameter (mm)**	**Location**	**T1 GRE unenh**	**N° T1 GRE unenh**	**T2 TSE FatSat**	**N° T2 TSE FatSat**	**DWI 800**	**N° DWI 800**	**Pattern art enhancement**	**N° Art**	**Portal enhancement**	**N° portal**	**Venous enhancement**	**N° venous**
2	2	16/22	Head/Body	HypoInt	2	HyperInt	2	HyperInt	2	Homogeneous	2	HyperInt	2	IsoInt	1
3	1	40	Head	HypoInt	1	Hyper/IsoInt	1	HyperInt	1	Rim	1	Hyper/IsoInt	1	IsoInt	1
10	1	13	Head	HypoInt	1	HyperInt	1	HyperInt	1	Homogeneous	1	HyperInt	1	Hyper/IsoInt	1
12	1	39	Tail	HypoInt	1	IsoInt	1	HyperInt	1	Rim	1	Hyper/IsoInt	1	IsoInt	1
13	2	16/11	Body/Tail	HypoInt	2	HyperInt	2	IsoInt	1	IsoInt	2	IsoInt	2	IsoInt	1
Sum	7				7		7		6		7		7		5

Disease-free time interval between primary treatment and recurrence ranged from 1 to 17 years (mean 7.5 years).

In 11 patients the pancreas was the only metastatic site. 3 patients also presented with lung metastases, in one case associated with liver metastases. 1 patient had a concomitant thyroid metastatic lesion.

**Figure 1:**
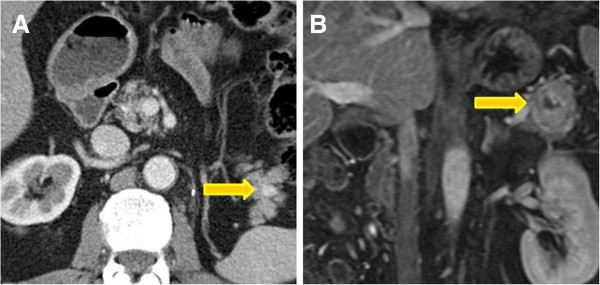
**Solitary lesions.** Two examples of solitary spherical tumors of the pancreatic tail, respectively on contrast-enhanced CT **(A)** and MRI **(B)** images, with the typical appearance of a predominantly solid, well-defined mass with smooth borders.

**Figure 2:**
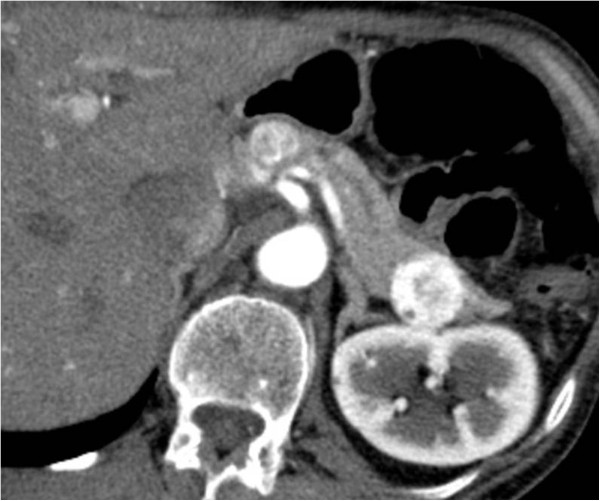
**Multiple lesions.** An arterial phase acquisition on CT shows two synchronous metastases as inhomogeneously enhancing nodular masses in the pancreatic body and tail.

Solitary lesions were found in 7 patients (Figure 1), while 8 patients presented with multiple metastases (Figure 2). The lesions were distributed throughout the pancreas without notable predilection for a particular part of the gland. No patients presented a diffuse involvement of the pancreas.

The metastatic foci usually appeared well-defined, often with discrete margins. 21 were round or ovoid with smooth borders, 5 were lobular. They ranged in size from 0.7 cm to 4.0 cm (median diameter was 1.5 cm).

In 2 patients there was evident obstruction of the main pancreatic duct with dilatation of the duct upstream from the obstruction. 4 cases presented biliary tree dilatation.

No patients demonstrated involvement of a neighbouring extra-pancreatic artery; even if venous involvement is often more difficult to assess, in our series the superior mesenteric and portal veins appeared unaffected.

**Figure 3:**
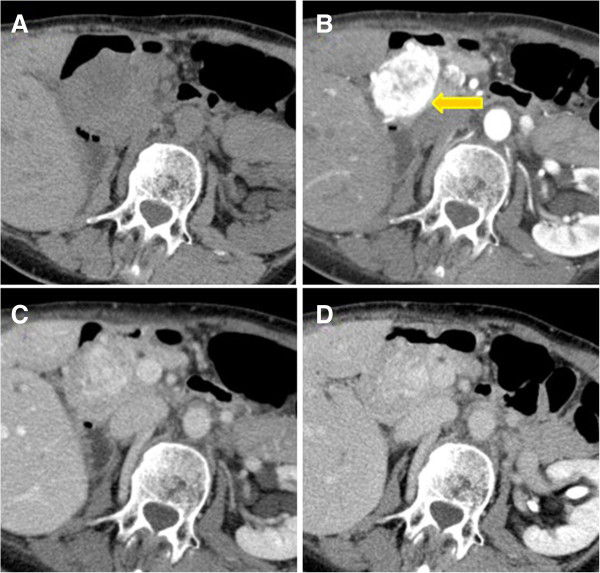
**Vascularity pattern.** Axial CT scans obtained unenhanced **(A)** and during arterial **(B)**, portal **(C)** and delayed **(D)** phases of contrast enhancement show a large, solitary round metastasis of the pancreatic head. Note that lesion is best seen in the arterial phase and less well seen in portal and delayed phases.

On contrast-enhanced CT, metastases avidly enhanced in the arterial or pancreatic parenchymal phase and demonstrated a significant wash-out on portal and delayed phase images, performed after 120 seconds (Figure 3). Nodules smaller than 2-2.5 cm in diameter showed homogeneous enhancement, while larger masses had an heterogeneous enhancement, with central hypodense areas probably due to necrosis.

**Figure 4:**
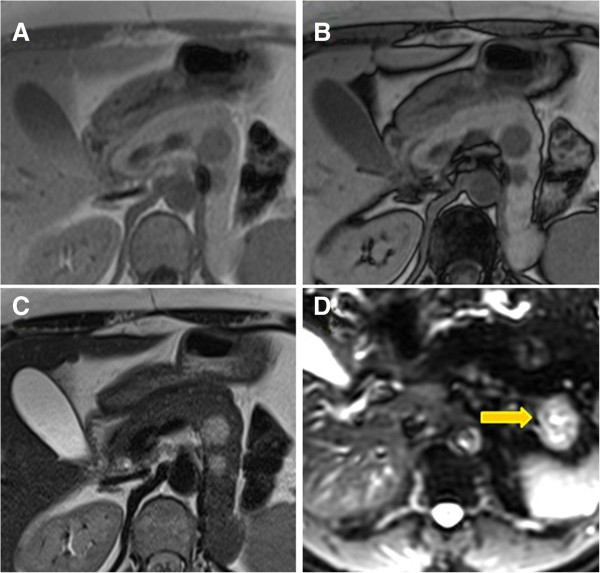
**In-phase, out-of-phase and diffusion-weighted images.** Lesions with signal intensity lower than normal pancreatic tissue on in-phase **(A)** and out-of-phase images **(B**). They showed hyperintense signal on T2-weighted **(C)** and diffusion-weighted sequences obtained at b values of 400 **(D)**.

On MRI, the lesions showed signal intensity lower than normal pancreatic tissue on pre-contrast T1-weighted images, both on in-phase and out-of-phase acquisitions (Figure 4). They conversely presented an heterogeneous or moderately hyperintense signal on T2-weighted images with or without fat-saturation, including diffusion-weighted sequences.

After the administration of gadolinium, nodules smaller than 2-2.5 cm in diameter showed homogeneous enhancement, while larger masses presented heterogeneous enhancement with central hypodense areas, probably due to internal necrosis.

**Figure 5:**
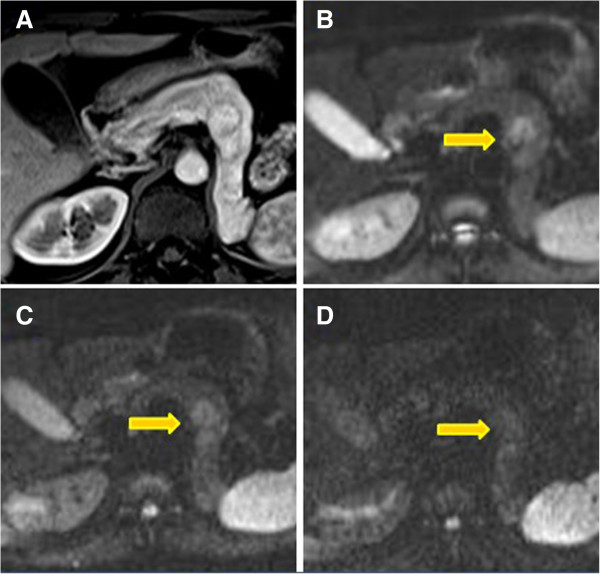
**Arterial phase and diffusion-weighted images.** Lesions almost isointense to the surrounding pancreatic parenchyma on arterial phase acquisition, circumscribed by a thin hypointense rim **(A)**. They show lower signal intensity on DWI sequences with the increasing of b-values **(B, C, D)**.

Only in 1 patient we found two metastatic foci that resulted almost isointense to the surrounding pancreatic parenchyma, from which they were separated by a thin hypointense rim. These lesions also showed loss of signal intensity on DWI with high b-values, while the other lesions in our series showed increased signal intensity with higher b-values (Figure 5).

## Discussion

Metastases from renal cell carcinoma (RCC) may be found at the time of primary tumour diagnosis or, more frequently, during follow-up after surgery [5,7,11]. A report showed that approximately 10% of 10-year survivors after tumor nephrectomy had late recurrences from RCC [5].

Nevertheless, pancreatic metastases from RCC are unusual. In autopsy series of patients with RCC, the reported incidence ranged from 1 to 3% [12].

If pancreatic metastases are discovered at the follow-up, pancreas not unfrequently is the only metastatic site and relatively often has a solitary metastasis. In fact, RCC has been reported to be the most common primary tumor leading to solitary pancreatic metastasis [13]. No relationship has yet been found between the site of an isolated pancreatic metastasis and the site of the primary renal cell carcinoma [14].

However, multifocality of pancreatic metastases from RCC is not unusual, ranging from 20% to 45% [3,15]. It does not necessarily relate to a worse outcome and it does not represent a contraindication to surgery.

Despite the uni- or multifocality, when there are no detectable metastases in other organs there is a favorable indication for a radical surgery, with a reported mean survival of 4 years [3]. In fact surgery is considered the gold standard therapy for localized disease.

In addition, the introduction of targeted therapy, including inhibitors of vascular endothelial growth factor (VEGF) and mammalian target of rapamycin (mTOR), has dramatically changed the outcome of patients with metastatic renal cell cancer, which was typically a chemoresistant disease [16].

For all these reasons, it is crucial to achieve an early radiological diagnosis of recurrence.

In our series, both on CT and MRI the metastatic foci appeared as round or ovoid masses, mostly well-delineated and with smooth borders.

As reported in the literature, metastases resulted isodense or hypodense in comparison to normal parenchyma on unenhanced CT, avidly enhanced in the pancreatic late arterial phase and demonstrated a significant wash-out, in particular on delayed phase images.

In the delayed phase lesions appeared sometimes hard to recognize, approaching isodensity with the surrounding pancreatic parenchyma: acquiring an arterial phase is therefore mandatory whenever we are dealing with a patient with history of RCC, whatever the clinical indication for imaging [2,4,12].

Upon our knowledge, there are few reports in the literature concerning MRI imaging features of pancreatic metastases from RCC.

In our experience, metastases showed low signal intensity compared with the normal pancreatic tissue on pre-contrast T1-weighted images, and moderately hyperintense signal on T2- and T2 fat-sat images. Lesions larger than 2 cm often had heterogeneous signal both on T1- and T2-weighted sequences, due to necrosis [17].

After the administration of gadolinium, metastases presented enhancement features similar to those described with CT. An early and homogeneous enhancement was usually seen in smaller metastases, while larger lesions showed a thick rim of enhancement.

In one patient, two intrapancreatic lesions appeared almost isointense to the surrounding pancreatic parenchyma on dynamic contrast enhanced MRI. This behavior, perhaps, might be related to a higher necrotic content of the lesions, as suggested also by the lowering of their signal intensity on DWI acquisitions with the increasing of the b-values, while the other metastatic lesions in our series showed a persistent elevated signal.

The non-enhanced features described above, and the early enhancement after contrast medium injection with both CT and MRI are characteristics that can also be found in the presence of endocrine pancreatic tumors, while primary adenocarcinomas tend to be hypovascular [18].

It is really hard to differentiate RCC metastases from endocrine tumors of the pancreas.

**Figure 6:**
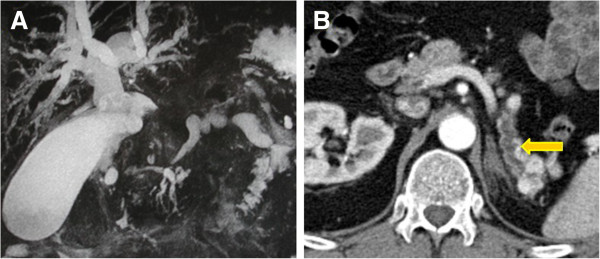
Obstruction of the common bile duct with upstream dilatation of the biliary tree (A); distal dilatation of the main pancreatic duct (B).

Symptoms of pancreatic metastases such as abdominal pain, jaundice, weight loss, and steatorrhea may be caused by tumoral invasion of the choledochus or main pancreatic duct (Figure 6). Pancreatic endocrine tumors usually do not invade these structures: this could help in the differentiation between pancreatic metastasis from RCC and endocrine tumor [7,19].

Moreover, a pancreatic endocrine tumor will cause endocrine symptoms if it is a functional tumor.

Even if octreotide or somatostatin receptor scintigraphy is helpful in the identification of neuroendocrine tumors, some metastatic lesions may also express somatostatin receptors, showing an intense uptake of octreotide at scintigraphy [20]. This should be kept in mind as it could mislead the diagnosis. On the other hand, it might result useful for therapeutic purposes.

Concerning primary adenocarcinomas of the pancreas, the fundamental clue for the differential diagnosis with RCC metastases consists in the pattern of enhancement after contrast medium, as described above. Also multifocality within the pancreas, relatively common in the presence of recurrence, is not characteristic of primary pancreatic carcinoma.

Another point of differentiation could be the evidence of encasement or infiltration of the peripancreatic arteries and veins. In our series no patients with metastases from RCC showed involvement of these structures.

However, the clinical history remains always the first thing to consider. An hypervascular pancreatic tumor in a patient with a previously diagnosed renal cancer should be considered a metastasis from renal cancer until proven otherwise.

## Conclusion

Pancreatic metastases may present many years after the resection of the primary renal cell carcinoma.

An early arterial phase or a pancreatic parenchymal phase are mandatory both on CT and MRI for patients with a history of RCC, despite the reason why the patient is undergoing the exam or the time passed after surgery.

With regard to the differential diagnosis, endocrine pancreatic tumors are the most challenging ones, as the other primary pancreatic tumors tend to be hypovascular.

However, clinical history remains extremely helpful. A hypervascular pancreatic tumor in a patient with a history of renal cancer should be considered a metastasis from renal cancer until proven otherwise.

## Competing interests

The authors declared that they have no competing interests.

## Authors’ contributions

VM has been involved in planning the study, in acquisition of data and radiological examinations. He has also performed the statistical analysis. PG drafted the manuscript and has made substantial contributions to conception and design. PR has participated in the design of the study helped to draft the manuscript. PC has followed patients for surgical and clinical aspects, giving important contributions to interpretation of data. PF have given final approval of the version to be published. All authors read and approved the final manuscript.
